# Clinical outcomes of bleomycin electrosclerotherapy (BEST) for the treatment of slow-flow vascular malformations (SFVMs): a UK single-centre experience

**DOI:** 10.1186/s42155-026-00688-6

**Published:** 2026-08-14

**Authors:** Nicolò Ubaldi, Arjan Singh Khattar, Mathew Storey, John Adu, Leto Mailli, Robert Morgan, Tobian Muir, Lakshmi Ratnam

**Affiliations:** 1 Department of Surgical, Medical Sciences and Translational Medicine, and Interventional Radiology, Azienda Ospedaliera Sant’Andrea, Sapienza University of Rome, Rome, Italy; 2 Department of Radiology, St. George’s University Hospitals NHS Foundation Trust, London, SW17 0QT UK; 3 School of Health & Medical Sciences, City St. George’s University of London, London, SW17 0RE UK; 4 Department of Plastic Surgery, James Cook University Hospital, Middlesbrough, TS4 3BW UK

**Keywords:** Bleomycin, Electrosclerotherapy, Low-flow vascular malformations, Slow-flow vascular malformations, Venous malformations, Lymphatic malformations, Sclerotherapy, Electroporation, Minimally invasive treatment

## Abstract

**Background:**

Bleomycin Electrosclerotherapy (BEST) is an emerging treatment for slow-flow vascular malformations (SFVMs), utilising electroporation to enhance intralesional bleomycin uptake. This study evaluates the safety and clinical outcomes of BEST in reducing symptom severity (SS) and improving quality-of-life (QoL) in patients with SFVMs.

**Methods:**

This retrospective, single-centre study analysed 20 consecutive patients with symptomatic SFVMs treated with BEST between September 2022 and November 2024. Patients underwent SS and QoL treatment specific questionnaires before treatment and at follow-up (mean: 286 days). Intralesional bleomycin injection was immediately followed by electroporation in every case.

**Results:**

A total of 42 BEST procedures were performed, on the following anatomical areas: Face: 14 (70%), Neck: 2 (10%), Leg: 3 (15%), Arm: 1 (5%). Significant reduction in the mean SS (-8.2, *p* < 0.001) and QoL (-5.2, *p* < 0.001) scores was observed. The most improved domains were swelling (mean -2, *p* < 0.001), aesthetic concerns (mean -2, *p* < 0.001), and pain (mean -1.1, *p* < 0.003). No major complications were reported. Skin changes (needle marks) were reported in 13/20, and intra-oral ulcers occurred in 6/20 patients. Most skin changes were either fading away or resolved at follow up. All intra-oral ulcers resolved during follow up.

**Conclusions:**

BEST is a safe procedure and demonstrates significant symptom relief and QoL enhancement, particularly in patients with severe baseline symptoms and more conspicuous lesions. While multiple treatments yielded greater improvements, even a single treatment was effective. Post-procedural side effects were minor, the most severe being skin markings and self-limiting ulceration.

**Supplementary Information:**

The online version contains supplementary material available at 10.1186/s42155-026-00688-6.

## Background

The first-line treatment for slow-flow vascular malformations (SFVM) as classified by the International Society for the Study of Vascular Anomalies is sclerotherapy, with surgery having a limited role [1, 2]. Bleomycin, a chemotherapy agent that induces endothelial cell death and vascular occlusion, is a commonly used and effective sclerosing agent. However, some malformations exhibit suboptimal response despite repeated treatment sessions due to limited endothelial cell uptake [3]. Using electroporation as an adjunct to bleomycin sclerotherapy has been shown to increase endothelial cell membrane permeability [4]. This increases the effectiveness of bleomycin by significantly increasing the cellular uptake [5]. Unlike conventional sclerotherapy, a general anaesthetic (GA) is usually required for bleomycin electrosclerotherapy (BEST), due to the delivery of the electric current which is painful.

BEST is particularly useful for malformations that are treatment-resistant, extensive or high risk for surgery, but its role may grow due to its increased effectiveness and potential to reduce the total number of treatment sessions required [6, 7]. The available literature describing its use and clinical outcomes is limited in numbers, optimal treatment protocols, and long-term data on recurrence [8–10]. This single-centre study aims to add to the literature on safety and clinical outcomes of the technique, as well as reporting our treatment protocol.

## Methods

### Study design

This is a single centre, retrospective study of 20 patients with SFVMs treated with BEST, between September 2022 and November 2024. Data collection was performed using electronic patient records as well as the Picture Archiving and Communication System. The diagnosis of SFVM was made based on clinical history, symptoms, clinical examination and imaging. The indications for BEST treatment were symptomatic lesions, which were felt to be large enough to require multiple treatments with conventional sclerotherapy to achieve satisfactory response. Exclusion criteria were patients with exclusively aesthetic concerns, Bleomycin allergy, pulmonary or renal dysfunction, epilepsy/seizures, and a cumulative dose of bleomycin > 80,000 units (U).

Ultrasound (US) was the primary imaging modality, with Magnetic Resonance Imaging (MRI) occasionally employed to confirm lesion extent. Cases were discussed in multidisciplinary team meetings involving interventional radiologists, dermatologists and surgeons to confirm suitability for BEST treatment.

### Bleomycin electrosclerotherapy (BEST)

All procedures were performed under GA. For intra-oral lesions, specific airway safety concerns were addressed with anaesthetists, and referring surgeons were involved in procedural and post-operative care planning. A bleomycin specific protocol was utilised to minimise side effects. The full protocol is provided as an example (Appendix 1).

All lesions were targeted percutaneously under US guidance. Intralesional contrast injection under fluoroscopy was then performed to exclude any deep venous drainage or arterial communication. A foamed mixture of bleomycin was injected until satisfactory coverage was confirmed with US. The foam mixture comprised of bleomycin (5000U in 5 ml), albumin (1 ml), Lignocaine (2 ml) and air (5 ml). Immediately after the injection, 2nd generation Series NFD, Wearable Finger Electrode electroporators (Model F-20-NL) with longitudinal configuration 20 mm needle length (CliniporatorTM VITAE, model EPS02, IGEA S.p.A., Carpi, Italy) were carefully applied over the treatment area in all cases. Needle electrodes were inserted following the injection trajectory and advanced to a depth of up to 2 cm, depending on lesion characteristics as assessed on pre-procedural US. Finger electrodes are of low voltage; the pulse amplitude ranges from 20 to 200 V with a pulse duration of 1 to 1000 ms. For larger lesions, multiple pulse deliveries were used, but no overlap is required.

### Follow-up

**Fig. 1:**
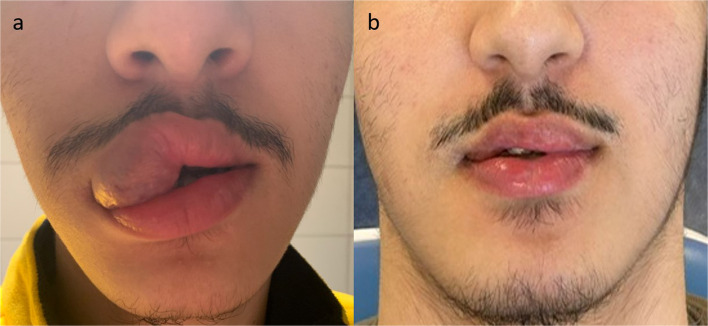
A 19-year-old male with a right upper lip slow flow venous malformation underwent 4 consecutive BEST sessions. a- Pre-treatment: Increase in lesion size over a few years with increased swelling (especially in dependent positions), bleeding and pain. Reports aesthetic concerns, discomfort and difficulties when eating. b- Post-treatment: Complete resolution of upper lip lesion. Minimal post-treatment symptoms on each occasion

**Fig. 2:**
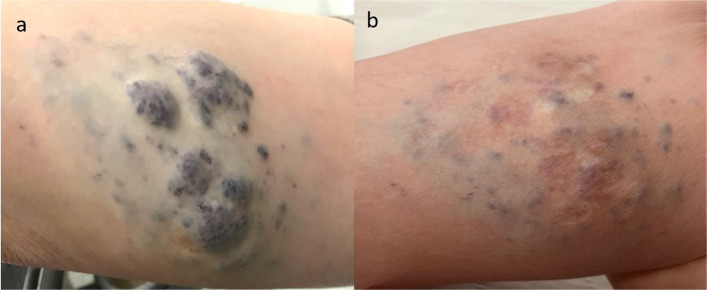
A 31-year-old male with a right arm slow flow venous malformation underwent 2 consecutive BEST sessions. a- Pre-treatment: Several sessions of conventional sclerotherapy as a teenager. Gradual increase in size and discoloration with worsening pain, swelling, discomfort and tenderness. b- Post-treatment: Reduction in size and asymptomatic

**Fig. 3:**
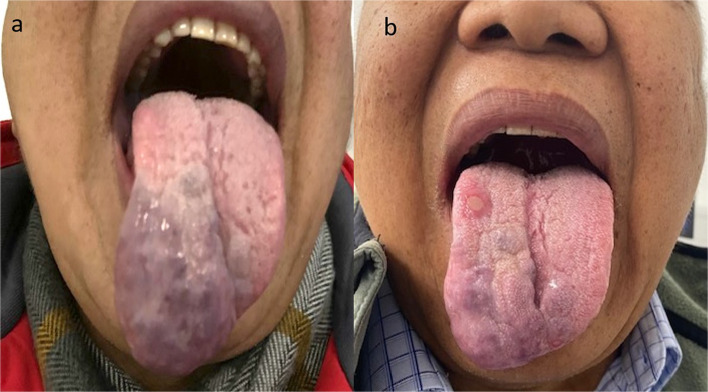
A 65-year-old male with a tongue slow flow venous malformation underwent 2 sessions of BEST. a-Pre-treatment: Increasing in size for approximately 8 years. Significant impairment of speech, swallowing and sleep. Multiple co-morbidities including previous stroke. b- Post-treatment: Significant size reduction after 2 sessions of BEST. Patient describes no impairment in speech, swallowing or sleep. Declined any further treatment

Two-month post-procedure ultrasound clinic follow up was conducted to assess clinical and imaging response. Repeat treatment was scheduled if needed (Figs. 1, 2, and 3). Bleomycin dose was recorded for each treatment and cumulative dose was monitored over multiple treatments to ensure that lifetime dose limit was not exceeded.

### Analysis and statistics

Statistical analysis was performed using open-source Python packages (Pandas and Matplotlib). The primary outcomes were total symptom severity score and quality of life measures, assessed using questionnaires developed from our extensive vascular malformation practice and a prior publication from our institution [11]. Statistically significant level was set at alpha = 0.05. The Wilcoxon Signed Rank test was used to assess the symptom scores (SS) and quality of life (QoL) burden pre- and post-procedure. The Kruskal–Wallis test was used to assess relationships between categorical variables and the post-procedural difference in SS and QoL. Spearman’s Rank correlation was used to assess relationships between continuous variables and the pre-procedural differences and QoL burden.

### Pre-procedural assessment of symptoms and quality of life

The treatment-specific, patient-reported questionnaires regarding SS and QoL were completed by 20/20 patients. The SS questionnaire included assessing 10 different parameters, ranging from a score of 0–5, with 5 being most severe. These parameters included: pain, swelling, concerns regarding appearance, changes in appearance over time, fatigue, bleeding, bruising, headaches, blurred vision and itchy skin. The QoL questionnaire included assessing 7 different parameters, ranging from a score of 0–5, with 5 being most negatively affected. These parameters were: mobility, daily activities, work-study, sports, leisure-playing, confidence-self-esteem and emotional wellbeing. Samples of the questionnaire are provided in Appendix 2.

## Results

### Patient population

A total of 42 BEST treatments were carried out for symptomatic SFVMs in 20 consecutive patients; 9 males and 11 females. Data documented included age, treated site and type of lesion, and number and type of previous treatments. These are described in Table 1. Both therapy-naïve patients (10/20, 50%), and patients who had undergone previous sclerotherapy were included. The mean follow-up interval was 286 days [39–629].

**Table 1:** Baseline population characteristics

Metric	Details
Total Patients	20
Total Number of Procedures	42
Age	Mean [range]: 35 years [7–66]
Gender Distribution	Female: 11 (55%), Male: 9 (45%)
Area of Body Affected	Face: 14 (70%), Neck: 2 (10%), Leg: 3 (15%), Arm: 1 (5%)
Vascular Malformation Subtype (in US Clinic)	Venous 13 (65%), Veno-lymphatic 5 (25%), Lymphatic (mixed; micro- and macro-cystic) 1 (5%), Haemangioma 1 (5%)
Patients' Expectations	Symptom improvement 15 (75%), Resolution 2 (10%), Change 2 (10%), None 1 (5%)
Patients’ Priority	Appearance: 8 (40%), Symptoms: 7 (35%), Function: 5 (25%)
Number with Previous Sclerotherapy	10 (50%)
Number of Previous Treatments	Mean [range]: 4.6 [2—12 treatments]
Previous Sclerotherapy	None: 10; Bleo + STS: 4, Bleo + Doxy + STS: 2, STS 3, Bleo only: 1
Total Treatment Interval (for those with multiple treatments)	Mean [range]: 296 days [70—588]

(Key: Bleo = bleomycin, STS = sodium tetradecyl sulfate, Doxy = doxycycline)

### Pre-procedure questionnaire

The SS domains and the number of patients affected pre-procedure were pain (14/20), swelling (19/20), cosmetic disfiguration (20/20), bruising (7/20), fatigue (5/20), bleeding (4/20), headaches (2/20), blurred vision (2/20) and pruritus (1/20).

The impaired QoL domains and the number of patients affected pre-procedure were confidence-self-esteem (15/20), emotional well-being (11/20), sport (10/20), daily activities (10/20), leisure/playing (8/20), work/study (6/20) and mobility (4/20). Baseline population characteristics are depicted in Table 1.

### Post-procedural characteristics

A summary of the total number of procedures, follow-up interval, total bleomycin dose and number of electrical pulses delivered are summarised in Table 2.

**Table 2:** Post-procedure bleomycin and electrode characteristics

Metric	Details
Number of BEST Treatments	Mean [range]: 2.1 [1–5]
Follow-Up Interval (Days)	Mean [range]: 286 [39—629]
Bleomycin Dose per Treatment (U)	Mean [range]: 3314 [1154—7000]
Electrodes per Treatment	Mean [range]: 6 [1–16]
Electrode Pulses over BEST Session	Mean [range]: 11.9 [1—34]
Total Bleomycin Dose over BEST Session (multiple treatments per patient) (U)	Mean [range]: 6960 [1154–17499]

(Key: A “Treatment” was defined as one individual procedure, while a “Session” referred to a series of consecutive “Treatments” performed in the same patient until an optimal response was achieved)

Complications were categorised using the Cardiovascular and Interventional Radiology Society of Europe classification system [12] (Table 3). The most frequent complications were pain and swelling, which were reported by 19/20 and 18/20 patients, lasting a mean of 8.7 and 10.6 days respectively. These are expected post-procedure treatment responses. There were no major complications (grade > 2). Skin changes (needle marks) were reported in 13/20 but improved in 6 patients and resolved completely in 2 patients during the follow-up period. No skin discoloration was observed; although patients were consented for this possible complication. Ulcers were reported in 6/20; all of these were intra-oral (2 on the tongue) and all 6 patients had transmucosal treatment inside the lips/cheeks or tongue. All of these ulcers resolved within a few weeks of the procedure. No pulmonary or renal complications occurred.

**Table 3:** Safety and complications

Side Effect/Complications	Number	Mean Duration (Days)
Off Work	17	5.3
Pain	19	8.7
Swelling	18	10.6
Difficulty Swallowing	3	1.9
Fever	0	0
Reduced Mobility	2	1.2
Bruising	3	1.9
Bleeding	2	0.5
Ulceration	6	2.5
Infection/Abscess	2	0.5
Altered Sensation/Tingling	5	2.7
Needle Marking	13	2 resolved; 6 fading

### Patient-reported questionnaire outcome

The SS domains and the number of patients affected post-procedure were pain (7/20), swelling (15/20), cosmetic disfiguration (13/20), bruising (5/20), bleeding (1/20), fatigue (3/20) headaches (1/20), blurred vision (1/20) and pruritus (0/20). The impaired QoL domains and the number of patients affected post-procedure were confidence-self-esteem (11/20), emotional well-being (10/20), sport (6/20), daily activities (8/20), leisure/playing (4/20), work/study (2/20) and mobility (2/20). Patient reported outcomes are summarised in Table 4.

**Table 4:** Patient reported outcomes

Patient Reported Effectiveness of Treatment	Number (%)
Excellent (70–90%)	13 (65%)
Moderate (50–70%)	6 (30%)
Mild (20–50%)	0
None (0–20%)	1 (5%)
Patient Satisfaction	
Yes	20 (100%)
No	0

### Comparative results

There was a mean 8.2 [0–25] point improvement in total SS (*p*-value < 0.001) following BEST (Table 5). The most pronounced improvement was in pain (mean −1.1, *p* = 0.003), swelling (−2.0, *p* < 0.001) and appearance (−2.0, *p* < 0.001). There was a mean 5.2 [0–16] point improvement in mean QoL burden (*p* < 0.001) (Table 6). The most pronounced improvement was in confidence/self-esteem (mean −1.4, *p* < 0.001), emotional wellbeing (−0.75 *p* = 0.020), sports (−0.70 *p* = 0.016), daily activities (−0.65 *p* = 0.047) and work-study (0.65, *p* = 0.042).

**Table 5:** Symptom Severity scores before and after procedure

Variable	Mean Diff. (Post—Pre)	Median Diff. (Post—Pre)	*p*-value
**Symptom Severity Score**	**−8.2**	**−7**	**< 0.001**
Pain	−1.1	−1	0.003
Swelling	−2.0	−2	< 0.001
Concerns regarding appearance	−2.0	−2	< 0.001
Changes in appearance over time	−1.7	−2	< 0.001
Fatigue	−0.3	0	0.059
Bleeding	−0.5	0	0.102
Bruising	−0.3	0	0.244
Headaches	−0.2	0	0.276
Blurred vision	0	0	1.000
Itchy skin	−0.1	0	0.317

The bold scores are the total score and the other components (not bolded) are the breakdown parameters of that score

**Table 6:** Quality of Life scores before and after procedure

Variable	Mean Diff. (Post—Pre)	Median Diff. (Post—Pre)	*p*-value
**Quality of Life Burden**	**−5.2**	**−4**	**< 0.001**
Mobility	−0.3	0	0.102
Daily activities	−0.7	−1	0.047
Work-study	−0.7	0	0.042
Sports	−0.7	−1	0.016
Leisure-playing	−0.8	0	0.017
Confidence/self-esteem	−1.4	−2	< 0.001
Emotional wellbeing	−0.8	−1	0.020

The bold scores are the total score and the other components (not bolded) are the breakdown parameters of that score

The sum of mean scores of all domains of SS and QoL improved from 22.5/85 to 8.9/85 (*p* < 0.001). Higher bleomycin doses or number of electrode pulses were not associated with higher improvements in SS and QoL scores (Table 7).

**Table 7:** Bleomycin dose and number of electrode pulses correlation with SS and QoL scores

Variable	Spearman Rank Correlation Coefficient (SRC)	*p*-value
Bleomycin per session vs Symptom Severity change	0.13	0.60
Bleomycin per session vs Quality of Life change	−0.18	0.45
Electrode pulses per session vs Symptom Severity change	0.08	0.75
Electrodes pulses per session vs Quality of Life change	−0.13	0.60

Key: A “Treatment” was defined as one individual procedure, while a “Session” referred to a series of consecutive “Treatments” performed in the same patient until an optimal response was achieved

Patients with recurrent or refractory lesions reported improvements with BEST comparable to those who were sclerotherapy naive (*p* = 0.5).

### Single vs. multiple procedures

Patients undergoing multiple procedures exhibited a greater improvement in SS, with a mean reduction of 9.5 points (64%—vs. 46% in the single-procedure group), and a mean QoL improvement of 4.7 points (56%—vs. 40% in the single-procedure group). These results were not significant (*p* > 0.05).

## Discussion

This study confirms the safety and reports the clinical outcomes of BEST treatment for SFVMs, obtaining significant improvements in patients’ SS (*p* < 0.01, Table 5) and QoL (*p* < 0.01, Table 6). According to specific patient questionnaires, more than half of the population reported a perceived > 70% treatment effectiveness. The most severe post-procedural complications were skin markings (electrode needle marks) [13]. However, in more than half of the patients they were fading away at follow up, with complete resolution in 2 (10%). Other post-procedure side effects were temporary, the most common of which were pain and swelling. Out of the 6 patients with tongue lesions, 2 presented with severe ulcers which were self-limiting. Corticosteroid mouthwash was helpful for symptom relief. The timing of these side effects is important for patient consent and advice in managing post-treatment clinical course. Our experience is that pain and swelling worsens at approximately the second day post-procedure and can last for up to 3 weeks. No major complications were encountered.

Sclerotherapy is the first-line treatment for SFVMs and bleomycin is a well-established effective sclerosant [3, 14, 15]. However, conventional sclerotherapy with bleomycin requires multiple procedures [14]. To enhance the effect of bleomycin, BEST utilises reversible electroporation, which creates temporary pores in cell membranes, facilitating increased drug absorption [16, 17] and greater clinical outcomes [18]. This procedure could be especially useful in lesions refractory to conventional sclerotherapy, microcystic lymphatic malformations, and larger lesions.

Although a relatively new technique, BEST has been found to be safe and effective for children and adults in a large retrospective multicentre cohort study [19], reporting aesthetic improvement in 64.6%, unchanged or improved pain in 88.5% with a major complication rate of 8.9%. Major complications reported by others included skin necrosis, persistent arterial spasm, nerve injury and disseminated intravascular coagulation. Bleomycin is also known for its risk of pulmonary toxicity when used systemically. However, localised administration minimises this risk and is very rare in sclerotherapy [19].

The cost of performing a single BEST procedure is significantly higher compared to that of a standard sclerotherapy session, mainly due to the need for a GA and the use of electrodes. Nonetheless, given that BEST can reduce the number of treatment sessions required [8–10, 19], it is possible that the overall cost per patient may ultimately be comparable in the long term. Also, the overall dose of intralesional bleomycin in BEST is generally lower compared to traditional sclerotherapy [19]. Early in our series, repeat treatments were scheduled at 2–3 months. However, our experience, mirrored by others, is that symptom improvement continues for several months before gradually declining [8–10, 19]. Therefore, extending treatment intervals can optimise outcomes, reduce costs, minimise unnecessary GAs, and lower cumulative bleomycin dose.

In this study, a single BEST procedure improved SS by 5.9 points (46%) and QoL by 6 points (40%), whereas multiple procedures led to a greater reduction in SS (9.5 points, 64%) and in QoL burden (4.7 points, 56%). Improvement in the SS and QoL are clear in our cohort of patients. The overall score improvement, based on the combined mean values of all SS and QoL domains, was significant (mean—13.4 points, *p* < 0.001). All SS domains significantly decreased after treatment (Table 5), with the mean overall difference decreasing by 8.2 points (*p* < 0.001).

The mean overall difference in QoL assessments decreased significantly by 5.2 points (*p* < 0.001) (Table 6). All domains of the QoL burden questionnaire were significantly reduced (Table 6) except for mobility which only showed a trend towards improvement (however most of our lesions were in the head and neck where mobility was unaffected). Two out of 6 patients with tongue lesions reported an augmented sense of taste after treatment.

**Fig. 4:**
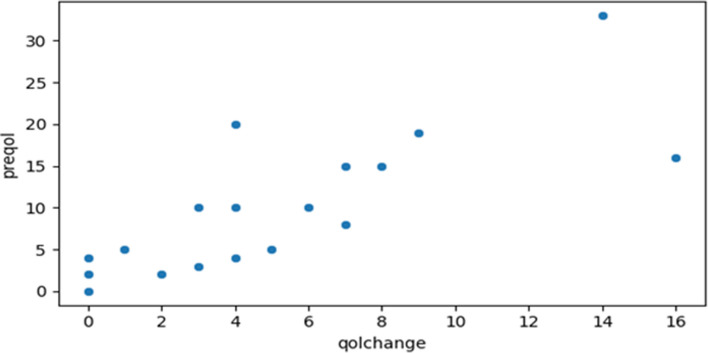
Correlation between baseline QoL and post-procedure QoL improvement

**Fig. 5:**
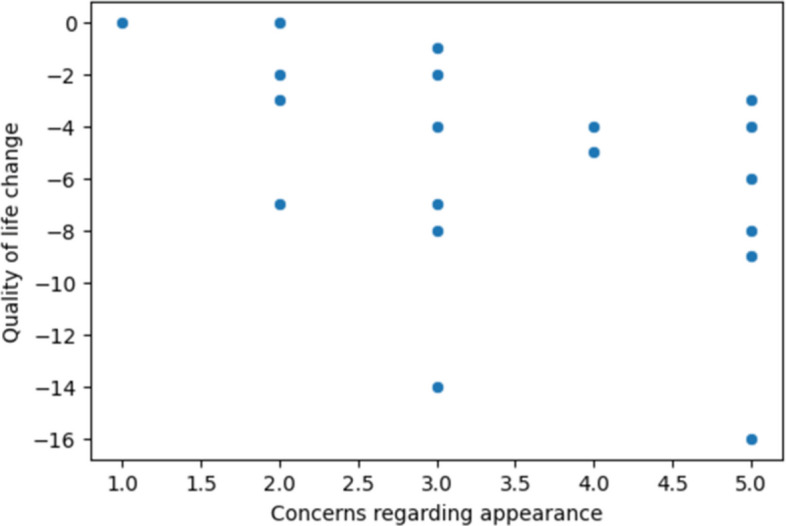
Correlation between baseline appearance concerns with QoL improvement post-procedure

Our findings suggest potential associations between baseline QoL severity and baseline appearance concern with the extent of benefit derived from the procedure, with more severely affected patients in both variables experiencing greater improvement in QoL (Figs. 4 and 5). Thus, it could be argued that patients with more severe baseline QoL and SS scores and greater aesthetic concerns should be prioritised for BEST as they may experience greater gains. Statistical power for these findings is limited by small sample size but this could be an avenue for future research.

Despite the procedure's low risk profile, its use should not be extended to patients with mild disease and small lesions (< 1 cm), as the risks associated with GA and chemotherapeutic agents, as well as the additional costs, may outweigh the benefits.

Patients with prior interventions initially had worse baseline QoL and SS, which remained worse post-procedure. However, the percentage improvement between the two groups did not significantly differ. In addition, our imaging findings are not correlated with SS or QoL, possibly due to suboptimal standardisation in the collection of imaging data. Size reduction has been reported following BEST [8, 19] and sclerotherapy [20].

Regarding limitations of this study, its retrospective nature and small population size reduce the strength and increase the bias of the results. Non-standardised imaging collection reduces the opportunities to find new useful correlations with treatment outcomes. Although the patient-reported questionnaires add important details to existing literature, they are not internationally validated to specifically assess BEST treatment. The post-procedure questionnaire was completed at different follow-up times from their last treatment between patients, increasing the heterogeneity of the results and our results were based on cumulative BEST procedure data rather than a single treatment. Additionally, no objective measurements of lesion size were made. This was deliberate given variation in lesion size and location and previous data [11] demonstrating that improvements in QoL and SS were the primary objectives for treatment. Selection bias for a new treatment modality is likely and it is important to remember that conventional sclerotherapy is very effective for most small vascular malformations and can be performed under local anaesthetic. More rigorous data is needed with regards to the type of lesion most likely to benefit from BEST with future research assessing classification, location and characteristics of malformations which will benefit most from this treatment.

## Conclusions

Bleomycin electrosclerotherapy is a new treatment that significantly improves symptom severity and quality of life. Even a single procedure achieved significant improvements. Patients should be informed that pain and swelling can be severe for up to 3 weeks post-procedure, and skin markings are common side effects although frequently temporary. Patients with more severe symptoms and more affected by the appearance of the lesions may derive more benefit from the procedure. Lastly, improvement in appearance correlated positively with enhanced quality of life. This paper adds real-world data on outcomes of this innovative treatment and supports its role in patient care.

## Supplementary Information

Supplementary Material 1: Appendix 1. Bleomycin Precautions Protocol.

Supplementary Material 2: Appendix 2. Questionnaires.

## Data Availability

The datasets used and/or analysed during the current study are available from the corresponding author on reasonable request.

## Abbreviations

SFVM: Slow-flow vascular malformation
GA: General anaesthetic
BEST: Bleomycin electrosclerotherapy
US: Ultrasound
MRI: Magnetic Resonance Imaging
SS: Symptom score
QoL: Quality of Life
