# Supplementary material for: Clinical outcomes of bleomycin electrosclerotherapy (BEST) for the treatment of slow-flow vascular malformations (SFVMs): a UK single-centre experience
Source: CVIR Endovasc. 2026 Aug 14;9:101. doi: 10.1186/s42155-026-00688-6 (PMC13473054; doi:10.1186/s42155-026-00688-6)
Supplement: Supplementary file 1 [file 42155_2026_688_MOESM1_ESM.doc]

**Local Interventional Radiology Bleomycin Anaesthetic Protocol**

Bleomycin is an antibiotic agent with cytotoxic activity. It is used as a sclerotherapy agent to shrink certain vascular malformation subtypes. Typically, 60% of an IV dose is excreted via kidneys unchanged; the remainder is mostly deactivated by the enzyme bleomycin-hydrolase in liver/spleen/bone marrow and gut. The skin and lungs lack this enzyme and bleomycin can accumulate here.

The risks of bleomycin exposure include

- pneumonitis (widely recognised but rare even after high dose IV administration; not reported yet with sclerotherapy)
- skin pigmentation

All reported with sclerotherapy

- nail discolouration
- alopecia

**Reducing the Risk**

**Risk of pneumonitis**

Far less likely if the patient is not exposed to supplemental oxygen at the time of bleomycin exposure or at any time in the future.

- Use the lowest oxygen concentration that is safe, air if possible.

**Skin marking**

Risk increased by trauma to any area of the skin while bleomycin is circulating in the bloodstream. There is no scientific data available to define/quantify risk. In some institutions that do few procedures skin marking has been quoted as high as 43%. At a London tertiary referral centre, since the introduction of these bleomycin precautions in 2015 to reduce the risks of skin marking, no further complications have been seen.

The risk is probably increased for 48 hours after injection. Markings are usually permanent and can be severe. They occur after excessive scratching and where anything adhesive has been pulled off the skin.

**Reducing the Anaesthetic risk**

- ETT – Tie the ETT rather than tape
- Eyes – Just Jelonet to keep them closed, no tape or lacrilube as patient rubs away.
- LMA – LMAs may not need securing, depending on how they sit and where the sclerotherapy injection site is
- ECG dots to be used with gel pads and placed in axillae and on sole of foot where possible. (If they do mark they would be in inconspicuous places)
- Cannula – secure with either mepitel or mepitac
- BP cuff – Velband (like cotton wool) should be used under cuff.
- Remove anything stuck to skin very carefully with Zoff or Appeel

**Post-op advice for theatre recovery and wards**

**for bleomycin sclerotherapy patients**

Bleomycin is used as a sclerotherapy drug to shrink some types of vascular malformation.

The side effects include

- chronic lung fibrosis (pneumonitis)
- marking of the skin with minor scratches or trauma. This can be permanent

Bleomycin circulates in the body for up to 48 hours after sclerotherapy. There are no scientific studies or published guidelines regarding the post operative management of patients to minimise these risks. We believe best practice should be:

- Avoid supplemental oxygen unless medically indicated. If you are concerned about the patient’s oxygen saturation, start the lowest amount of oxygen required to maintain their saturations and seek advice from a doctor immediately.
- Stick nothing to the skin
- Anything stuck to the skin should be very carefully removed with an adhesive solvent such as Appeel or Zoff
- Keep Velband (like cotton wool) between blood pressure cuff and skin
- After removing a cannula, do not apply a dressing. Just apply gentle pressure to the site until it stops bleeding
- Remind patient not to scratch any area of skin for 48 hrs. If the patient feels itchy, please contact doctors immediately to write up anti-itching medication.
- Urine and other body fluids can be disposed of normally
