# Supplementary material for: Clinical outcomes of bleomycin electrosclerotherapy (BEST) for the treatment of slow-flow vascular malformations (SFVMs): a UK single-centre experience
Source: CVIR Endovasc. 2026 Aug 14;9:101. doi: 10.1186/s42155-026-00688-6 (PMC13473054; doi:10.1186/s42155-026-00688-6)
Supplement: Supplementary file 2 [file 42155_2026_688_MOESM2_ESM.docx]

**(1) Please select your gender**

Male

Female

Other

**(3) PRESENTING SYMPTOMS**

**When did you first start noticing this lesion?**

**(2) Which area of your body is affected?**

Head and Neck

Trunk

Lower Limb

Upper Limb

Groin

Other (specify): …………………………

**………………………………………………………….**

**EVALUATION QUESTIONNAIRE**

**FOR TREATMENT OF LOW FLOW**

**VENOUS AND LYMPHATIC**

**MALFORMATIONS WITH**

**PERCUTANEOUS SCLEROTHERAPY**

**FIRST PRESENTION QUESTIONNAIRE	(PRE**

**-**

**TREATMENT**

**)**

**PATIENT'S SHEET**

**HOSPITAL NUMBER:**

**…………………**

**.**

**…**

**....**

**PATIENT'S NAME: .....................................**

**DATE OF REVIEW:**

**……………**

**.**

**……………**

**.**

**(4) Which of these are the reasons for your visit today? (Tick all that apply)**

 Pain

 Swelling

 Concerns regarding appearance

 Change in lesion over time

 Bleeding

 Bruising

 Headaches

 Blurred vision

Itchy skin

Fatigue

 Increase in size or discomfort with hot weather

 Recurrence of lesion or symptoms after treatment, please specify when was the last treatment………………………

Other ………………………………………………………

**(5) SEVERITY OF SYMPTOMS:**

**For the reasons selected above, please rate how distressing these symptoms are on a scale of 1 to** **5 where five is “most distressing” and 0 is “no concern at all’’**

|  | 0 | 1 | 2 | 3 | 4 | 5 |
| --- | --- | --- | --- | --- | --- | --- |
| Pain |   Does not hurt |   Hurts a  Little |   Hurts a little more |   Hurts even more |   hurts  a lot |   Hurts worse |
| Swelling |  |  |  |  |  |  |
| Concerns regarding Appearance |  |  |  |  |  |  |
| Change in Appearance over time |  |  |  |  |  |  |
| Fatigue |  |  |  |  |  |  |
| Bleeding |  |  |  |  |  |  |
| Bruising |  |  |  |  |  |  |
| Headaches |  |  |  |  |  |  |
| Blurred Vision |  |  |  |  |  |  |
| Itchy skin |  |  |  |  |  |  |

(OVAMA consensus, 2018)

(6) **OVERALL QUALITY OF LIFE:**

**Please if this lesion is affecting the overall quality of your life, would you specify by**

**choosing and rating the effect on a scale of 1 to 5 where five is “most distressing” and 0 is “no concern at all”.**

|  | 0 | 1 | 2 | 3 | 4 | 5 |
| --- | --- | --- | --- | --- | --- | --- |
| Mobility |  |  |  |  |  |  |
| Activities of Daily living |  |  |  |  |  |  |
| Work/Study |  |  |  |  |  |  |
| Sports |  |  |  |  |  |  |
| Leisure/Playing |  |  |  |  |  |  |
| Confidence/self-esteem |  |  |  |  |  |  |
| Emotional Wellbeing |  |  |  |  |  |  |

(OVAMA consensus, 2018)

(7) **PATIENT EXPECTATIONS:**

**Please describe your expected outcome following this treatment**

……………………………………………………………………………………………………………………………

…………………………………………………………………………………………………………..……………….

**How many sessions do you think this treatment will include?**

……………………………………………………………………………………………………………………………..

**Which of the following treatment outcomes would be your priority?**

 Reduction of symptom severity

 Improvement of cosmetic appearance

 Improvement of the function (e.g. if it is affecting your mobility, functioning or work)

**Do you expect recurrence of this lesion or your symptoms after treatment?**

 Yes

 No

 Do not know

(1) **RECOVERY PERIOD:**

**Please identify the length of your recovery period after the last procedure, and whether you had any of the following the responses. Please specify the duration of each:**

 Length of recovery period, till return to full activity/ work	(___ days)

 Pain	(___ days)

 Swelling	(___ days)

 Difficulty swallowing	(___ days)

 Fever	(___ days)

 Reduced mobility	(___ days)

 Others (please specify): …………………………………………

(2) **COMPLICATIONS:**

| **EVALUATION QUESTIONNAIRE**  **FOR TREATMENT OF LOW FLOW**  **VENOUS AND LYMPHATIC**  **MALFORMATIONS WITH**  **PERCUTANEOUS SCLEROTHERAPY** | **HOSPITAL NUMBER: ………………….…....**    **PATIENT'S NAME: .....................................**    **DATE OF REVIEW: …………….…………….** |
| --- | --- |
| **FOLLOW UP QUESTIONAIRE**  **PATIENT’S SHEET** | **POST-PROCEDURE**  **(SESSION.........., TREATMENT.........)** |

**Please identify if you experienced any type of complication following the last treatment and its duration**

 Bleeding	(___ days)

 Ulceration	(___ days)

 Infection/Abscess	(___ days)

 Altered sensation	(___ days)

 Other (please specify): ………………………………

**Had you experienced any complication, please specify whether you underwent treatment for it**

| **EVALUATION QUESTIONNAIRE**  **FOR TREATMENT OF LOW FLOW**  **VENOUS AND LYMPHATIC**  **MALFORMATIONS WITH**  **PERCUTANEOUS SCLEROTHERAPY** | **HOSPITAL NUMBER: ………………….…....**    **PATIENT'S NAME: .....................................**    **DATE OF REVIEW: …………….…………….** |
| --- | --- |
| **FOLLOW UP QUESTIONAIRE**  **PATIENT’S SHEET** | **POST-PROCEDURE**  **(SESSION.........., TREATMENT.........)** |

………………………………………….………………………………………..……………………………………..…

……………………………………………………………………………………………….…………………………… ………………………………………….………………………………………..……………………………………....

………………………………………………………………………………………………….…………………………

(3) **EFFECTIVENESS OF TREATMENT:**

**Please describe the degree of improvement you feel in your symptoms since the treatment has been started**

 Excellent Response (70%- 90% improvement)

 Moderate Response (50%- 70% improvement)

 Mild Response (20%- 50% improvement)

 No Response (0- 20% improvement)

(4) **PATIENT’S SATISFACTION:**

**Could you identify to what extent are you satisfied with the treatment response? In other words, “What are the chances that you would take this treatment again?”**

 Satisfied

 Dissatisfied

(5) **PATIENT EXPECTATIONS:**

**Did the explanation and the leaflet you received following the first consultation gave you enough information about the treatment plan, outcomes and possibility of recurrence?**

 Yes

 Somewhat

 No

**Is there anything you wish you had been told that you weren’t?**

……………………………………………………………………………………………………………………………

……………………………………………………………………………………………………………………………

(6) **SEVERITY OF SYMPTOMS:**

**Please rate how distressing your symptoms are, if you still have them, on a scale of 1 to 5 where five is “most distressing” and 0 is “no concern at all’’**

|  | 0 | 1 | 2 | 3 | 4 | 5 |
| --- | --- | --- | --- | --- | --- | --- |
| Pain |   Does not hurt |   Hurts a little |   Hurts a little more |   Hurts even more |   hurts  a lot |   Hurts worse |
| Swelling |  |  |  |  |  |  |
| Concerns regarding Appearance |  |  |  |  |  |  |
| Change in Appearance over time |  |  |  |  |  |  |
| Fatigue |  |  |  |  |  |  |
| Bleeding |  |  |  |  |  |  |
| Bruising |  |  |  |  |  |  |
| Headaches |  |  |  |  |  |  |
| Blurred Vision |  |  |  |  |  |  |
| Itchy skin |  |  |  |  |  |  |

(OVAMA consensus, 2018)

(7) **Overall Quality of Life improvement:**

**Please if this lesion is still affecting the overall quality of your life, would you specify by choosing and rating the effect on a scale of 1 to 5 where five is “most distressing” and 0 is “no concern at all”.**

|  | 0 | 1 | 2 | 3 | 4 | 5 |
| --- | --- | --- | --- | --- | --- | --- |
| Mobility |  |  |  |  |  |  |
| Activities of Daily living |  |  |  |  |  |  |
| Work/Study |  |  |  |  |  |  |
| Sports |  |  |  |  |  |  |
| Leisure/Playing |  |  |  |  |  |  |
| Confidence/self-esteem |  |  |  |  |  |  |
| Emotional Wellbeing |  |  |  |  |  |  |

(OVAMA consensus, 2018)
